# INTERNATIONAL INVESTIGATIONS OF MENTAL HEALTH AND PSYCHOSOCIAL WELL-BEING IN LATER LIFE

**DOI:** 10.1093/geroni/igae098.0922

**Published:** 2024-12-31

**Authors:** Giorgio Di Gessa, Brian Beach, Matthew Prina

**Affiliations:** Chair; Co-Chair; Discussant

## Abstract

Good mental health and psychosocial wellbeing are important factors for older individuals’ fortitude as they move through later life. Attention to mental health and wellbeing has continued to grow in recent years, with the COVID-19 pandemic playing a key role in raising the profile of psychosocial experiences across societies. However, many countries face ongoing challenges to provide support services that meet existing and projected needs. While these issues are not limited to older adults, people in later life may face vulnerabilities that make them more susceptible to negative outcomes in mental health and wellbeing or that introduce additional barriers to accessing adequate supports. This symposium highlights international investigations into mental health and wellbeing that enrich our understanding of these issues and some of the dynamics for identifying potential solutions to enhance support. The first presentation highlights the experience of older people’s psychosocial wellbeing across the pandemic using the English Longitudinal Study of Ageing (ELSA), with measures before, during, and in the years after the pandemic. The second presentation explores the concept of resilience in later life drawing on qualitative interviews among older people with long COVID from other longitudinal UK studies. The third presentation examines the role that mental health diagnoses play in shaping labor market exit among adults aged 65+ in paid work, drawing on Swedish register data. The final presentation investigates joint trajectories in depression scores and various blood-based biomarkers using ELSA data, exploring the potential for such measures to be useful in identifying risk for depression. International Comparisons of Healthy Aging Interest Group Sponsored Symposium

